# Neurotoxic non-protein amino acids in commercially harvested Lobsters (*Homarus americanus* H. Milne-Edwards)

**DOI:** 10.1038/s41598-024-58778-1

**Published:** 2024-04-05

**Authors:** Pawanjit K. Sandhu, Julia T. Solonenka, Susan J. Murch

**Affiliations:** Department of Chemistry, University of British Columbia, Syilx Okanagan Nation Territory, Kelowna, BC V1V 1V7 Canada

**Keywords:** Lobster, β-methylamino-alanine, Non-protein amino acid, Neurotoxin, New Brunswick, Natural hazards, Risk factors, Chemistry

## Abstract

Cyanobacteria produce neurotoxic non-protein amino acids (NPAAs) that accumulate in ecosystems and food webs. American lobsters (*Homarus americanus* H. Milne-Edwards) are one of the most valuable seafood industries in Canada with exports valued at > $2 billion. Two previous studies have assessed the occurrence of β-N-methylamino-L-alanine (BMAA) in a small number of lobster tissues but a complete study has not previously been undertaken. We measured NPAAs in eyeballs, brain, legs, claws, tails, and eggs of 4 lobsters per year for the 2021 and 2022 harvests. Our study included 4 male and 4 female lobsters. We detected BMAA and its isomers, N-(2-aminoethyl)glycine (AEG), 2,4-diaminobutyric acid (DAB) and β-aminomethyl-L-alanine (BAMA) by a fully validated reverse phase chromatography—tandem mass spectrometry method. We quantified BMAA, DAB, AEG and BAMA in all of the lobster tissues. Our quantification data varied by individual lobster, sex and collection year. Significantly more BMAA was quantified in lobsters harvested in 2021 than 2022. Interestingly, more BAMA was quantified in lobsters harvested in 2022 than 2021. The concentrations of BMAA we observed in lobsters are lower than an acutely toxic dose, but given previous research which has demonstrated that chronic exposure to low levels of BMAA can cause neurological abnormalities, we propose continued monitoring of lobster harvests for cyanobacterial neurotoxins to assess potential risks to human health.

## Introduction

Non-protein amino acids (NPAAs) are a class of naturally occurring metabolites with similar chemical structures to amino acids, the building blocks of proteins. The number of NPAAs produced in nature is unknown but it has been estimated that there are ≈900 NPAAs^1^. β-N-methylamino-L-alanine (BMAA) is an NPAA that can accumulate in ecosystems and food webs^2–4^. BMAA was first discovered in 1967 in seeds collected from *Cycas micronesica* Hill in Guam^5,6^. The original study was searching for an environmental cause to explain a cluster of cases of a neurological disease called Amyotrophic Lateral Sclerosis/Parkinsonism-dementia Complex (ALS-PDC) in Guam^7,8^. A symbiotic *Nostoc* isolated from roots of the Cycads^2^ was found to be the origin of BMAA^2,4^ and the NPAA is distributed through the plants to leaves and seeds^4^. The indigenous Chamorro people in Guam processed Cycad seeds into a flour used in tortillas and to thicken soups and sauces^2,9^. Animals that eat Cycad seeds, for instance flying foxes, deer and pigs in Guam^9^, are a secondary route of human exposure to BMAA^2,9–11^. Elsewhere in the world, BMAA is produced by cyanobacteria ^2,12–16^ and accumulated in plants, animals and seafood including; fish^17^, shellfish^18,18–21^, sharks^22–24^, and dolphins^25^, especially in places where cyanobacterial blooms are occurring^26^. Concerns about the health impacts of BMAA exposure were raised when it was identified and quantified in autopsy samples of patients who died of neurodegenerative diseases in Guam^11^, Florida^27,28^, Sweden^29^ and Canada^11^. Some studies did not detect BMAA in autopsy samples^30,31^, potentially as a result of methodological problems^3,32^. Neurotoxicology studies have demonstrated that exposure to BMAA can cause acute neurological injury^33^, neurofibrillary tangles and plaques in primates^34,35^, injury to motor neurons in cell cultures^36^, and motor neuron disfunction in a mouse model^37^. Detection and quantification of BMAA is complicated by the presence of natural isomers, complex sample matrices, difficulties in detection of low level metabolites and variability between methods^32,38^. The majority of the literature now uses well established and validated analytical methods^39–41^ and a recent meta-analysis of the whole scientific literature implicates BMAA as the leading potential environmental factor associated with ALS^42^.

Two previous studies have reported BMAA in American lobster (*Homarus americanus*) harvested in Canada^21^ and USA^20^ but a full study of the metabolism of BMAA and its naturally occurring isomers in lobsters has not been conducted. Our overall objective was to understand the distribution of BMAA and other non-protein amino acids: N-(2-aminoethyl)glycine (AEG), 2,4-diaminobutyric acid (DAB) and β-aminomethyl-L-alanine (BAMA) in whole lobsters to assess the potential risk of human exposure. We analyzed lobsters harvested, processed and sold commercially through the regular Canadian grocery market. Our data show that the amounts of BMAA in lobsters is variable by harvest year and suggests that the commercial lobster harvest should be monitored for BMAA accumulation in years or regions with active cyanobacterial blooms.

## Materials and methods

### Chemicals and standards

#### Standards

BMAA (CAS No. 16012-55-8, Sigma Aldrich); N-(2-aminoethyl)glycine (AEG) (CAS No. 24123-14-6, Sigma Aldrich); 2,4-diaminobutyric acid (DAB) (CAS No. 1883-09-06, TCI America); and in house custom synthesis of β-aminomethyl-L-alanine (BAMA)^15^.

#### Chemicals

Acetonitrile (CAS No. 75-05-8, Optima® LC/MS grade, Fisher Chemical, Mississauga, ON), Methanol (CAS No. 67-56-1, Optima® LC/MS grade, Fisher Chemical, Mississauga, ON), Formic acid (CAS No. 64-18-6, Optima® LC/MS grade, Fisher Chemical, Mississauga, ON), Ultrapure Water (18.2 MΩ cm, Direct Q3, Millipore, Mississauga, ON), 0.1N Trichloroacetic acid (TCA) was made by dissolving TCA reagent (> 99.0%, CAS No. 76-03-9; Sigma-Aldrich) in ultrapure water, Ammonium formate (> 99.995%, CAS No. 540-69-2, Sigma-Aldrich), 6N Hydrochloric acid (HCl, CAS No. 7732-18-5, 7647-01-0, Fisher Chemical, Mississauga, ON), 0.2M Borate Buffer and 6-Aminoquinolyl-N-Hydroxysuccinimidyl Carbamate (AQC) (AccQ Tag™ Ultra Derivatization Kit, Part no. 186003836, Waters, Mississauga, ON; kit reconstituted as per manufacturer instructions).

### Preparation of standards for calibration curve and derivatization

Standards were prepared using initial stock solutions made at 65 µmol/mL NPAA in 0.1N TCA. Three tenfold dilutions were performed, preparing concentrations of stock solutions at 0.065 µmol/mL for each isomer. Standard 1 was prepared by combining 100μL of each 0.065 µmol/mL NPAA stock solution together with 600μL of 0.1 N TCA. From this, a 6-step serial dilution series (Standards 2–7) was prepared from the higher concentration stock by taking 250µL of the previous standard and combining it with 750µL of 0.1 N TCA to create the next standard. Standards 2–7 were used for making a calibration curve and stored at −20 °C. For analysis, 20μL of each standard (2–7) was diluted with 60μL 0.2 M borate buffer (pH 10) in an autosampler vial (2 mL amber glass with pre-slit Teflon-coated caps; Waters Corp.) fitted with a conical bottom spring insert (250µL glass; Canadian Life Science, Peterborough, ON, Canada) and derivatized with 20μL of AQC tag followed by vortex mixing (Vortex Genie 2; Scientific Industries, Bohemia, USA) and incubation at 55 °C for 10 min to complete the reaction.

### Sample preparation

Commercially prepared lobster (*H. americanus;* ‘*Atlantic Star’* Royal Star Foods Ltd., Prince Edward Island, Canada) were sourced from a local grocery store. Lobsters were harvested from the Major Fishing Area 21 (Northwest Atlantic) (FAO, 2023) on 17 May 2021 and 26 May 2022, cooked and frozen on the vessel at sea. Samples obtained from the 2021 harvest consisted of three males and one female lobster and the 2022 samples consisted of three females and one male lobster. The frozen lobsters were removed from shells and sectioned into tissues and organs including, tails, claws, brain, eyeballs, legs, and eggs (for females). 100–200 mg from each tissue was weighed into a 1.5mL microcentrifuge tube (Fisher Scientific, USA) and homogenized with 2000μL of 0.1N TCA sequentially (1000μL twice) ((Kontes Pellet Pestle; Fisher) and vortexed at full speed for 30 s. Homogenized lobster samples were centrifuged (5 min. at 13,000 rpm) to pellet the proteins. An aliquot of 800μL of the supernatant was filtered through a Ultrafree®– MC – GV centrifugal filter tube (Durapore® 0.22 µm PVDF membrane; Merck Millipore) by centrifugation (5 min. at 13,000 rpm) to yield the filtered free amino acid extract. The protein pellet was hydrolyzed with 1000μL of 6N HCl in a glass hydrolysis vial (15 × 45 mm; Fisher Scientific), purged with nitrogen gas for 30 s, at 110 °C for 18–22 h on a heating block (VWR Standard Dry Block Heater). After cooling, 800µL of sample was filtered by centrifuging (Ultrafree®-MC PVDF) for 5 min at 13,000 rpm*.* 40 µL aliquot of the filtrate was dried to complete dryness (Speedvac, Labconco Centrivap, VWR). The dried filtrate was reconstituted with 100μL 0.2M borate buffer (Waters). 20μL of the reconstitute or free amino acid extract was derivatized with 20μL of AQC tag similar to standards.

### Amino acid analysis

Detection and quantification of BMAA, AEG, DAB and BAMA was performed by UPLC-MS/MS (Waters Acquity I-Class BSM, SM-FTN, column heater/cooler; Waters Xevo TQ-S with ESCi probe, MassLynx 4.1) as per previously published validated analytical procedures^16,39,44^. In brief, 10 μL aliquots of derivatized samples or standards were injected onto a reverse phase Acquity UPLC® BEH C_18_ column (2.1 × 100 mm; 1.7 µm, Waters) with temperature set to 55°C. Amino acids were eluted with a gradient-elution of 20 mM ammonium formate at pH of 5.0 adjusted with formic acid (Solvent A) and methanol (Solvent B) at a flow rate of 0.3 mL/min as follows: Initial: 90% A: 10% B; 7.0 min: 50% A: 50% B; 7.5 min: 25% A: 75% B; 9.6 min: 90% A: 10% B; 12 min: 90% A: 10% B. The needle and seal wash solvent consisted of 90% Acetonitrile: 10% ultrapure water. The ions were detected in positive mode with electrospray ionization (ESI) and multiple reaction monitoring (MRM) (Table 1 & Supplemental:^15,45^). The MS parameters were as follows: capillary voltage: 4.75 kV; cone voltage: 8.00 V; source offset: 20.0 V; source temperature: 150 °C; desolvation temperature: 550°C; cone gas (N_2_): 150 L/hr; desolvation gas (N_2_): 800 L/hr; collision cell flow (Argon): 0.30 mL/min; nebulizer gas flow: 7.00 bar. Peaks were detected and measured in chromatograms using TargetLynx v4.1 (Waters)^15,45^.

**Table 1:** Detection and performance characteristics for detection and quantification of non-protein amino acids: β-N-methylamino-L-alanine (BMAA), N-(2-aminoethyl)glycine (AEG), 2,4-diaminobutyric acid (DAB) and β-aminomethyl-L-alanine (BAMA) in American lobster (Homarus americanus). %RSD refers to relative standard deviation and MLOD is method limit of detection of the instrument.

Analyte	Retention Time (RT; min)	%RSD (RT)	Transition (m/z)	Cone Voltage (V)	Collision Voltage (V)	%RSD (signal)	MLOD (IUPAC)	% Recovery
BAMA	5.12	1.3	459 > 171 459 > 119 459 > 289	16 16 16	30 18 15	13.7	0.01	46.3
AEG	5.56	1.1	459 > 171 459 > 119 459 > 289 459 > 214	16 16 16 16	30 18 15 20	10.9	0.01	41.3
BMAA	5.78	1.2	459 > 171 459 > 119 459 > 289 459 > 258	16 16 16 16	30 18 15 20	18.7	0.005	68.8
DAB	6.07	1.2	459 > 171 459 > 119 459 > 289 459 > 188	16 16 16 16	30 18 15 20	20.6	0.01	53.0

### Method performance characteristics

The performance of analytical method was validated on linearity of calibration curves, percentage relative standard deviation (RSD), spike recovery and method limit of detection of the instrument. The respective information for each of the NPAA is given in Table 1 and supplemental information (Figure S1: S3, Table S1, S2).

### Statistical analysis

Each tissue was analyzed in triplicate extraction (*n* = 3) and there were 4 lobsters per year for 2021 and 2022. Across all years, there were 4 female and 4 male lobsters. Data were exported from TargetLynx to Excel for statistical analyses. Differences between treatments were determined by Student’s T-Test using the Excel protocol with *p* < 0.05. Data figures were created in RStudio (version 2023.03.0 + 386) using ggplot2 (version 3.4.2) and R (version 4.2.3).

## Results

**Figure 1:**
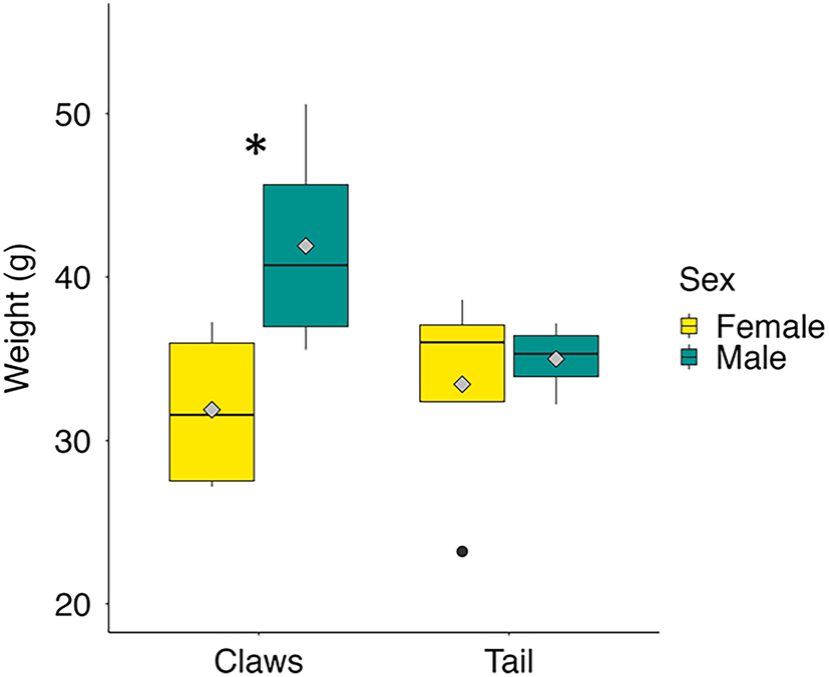
Distribution of weight (g) of tails and claws in male and female American lobster (*Homarus americanus*) collected over two harvest seasons. The diamond within each boxplot shows the mean weight. Asterisk denotes significant statistical differences (student’s t-test) at *p* < 0.05 (*n* = 4).

Lobsters were sourced from the commercial harvests of 2021 and 2022 through a local grocery store and were prepared in the same manner as for normal consumption. The tails were twisted and pulled away from the body to separate the meat. Claws were twisted away from the body and cracked to reveal the contents. Male lobsters had slightly larger claws than female lobsters (Fig. 1). A heavy knife was used to split the lobsters for access to the organs and tissues.

**Figure 2:**
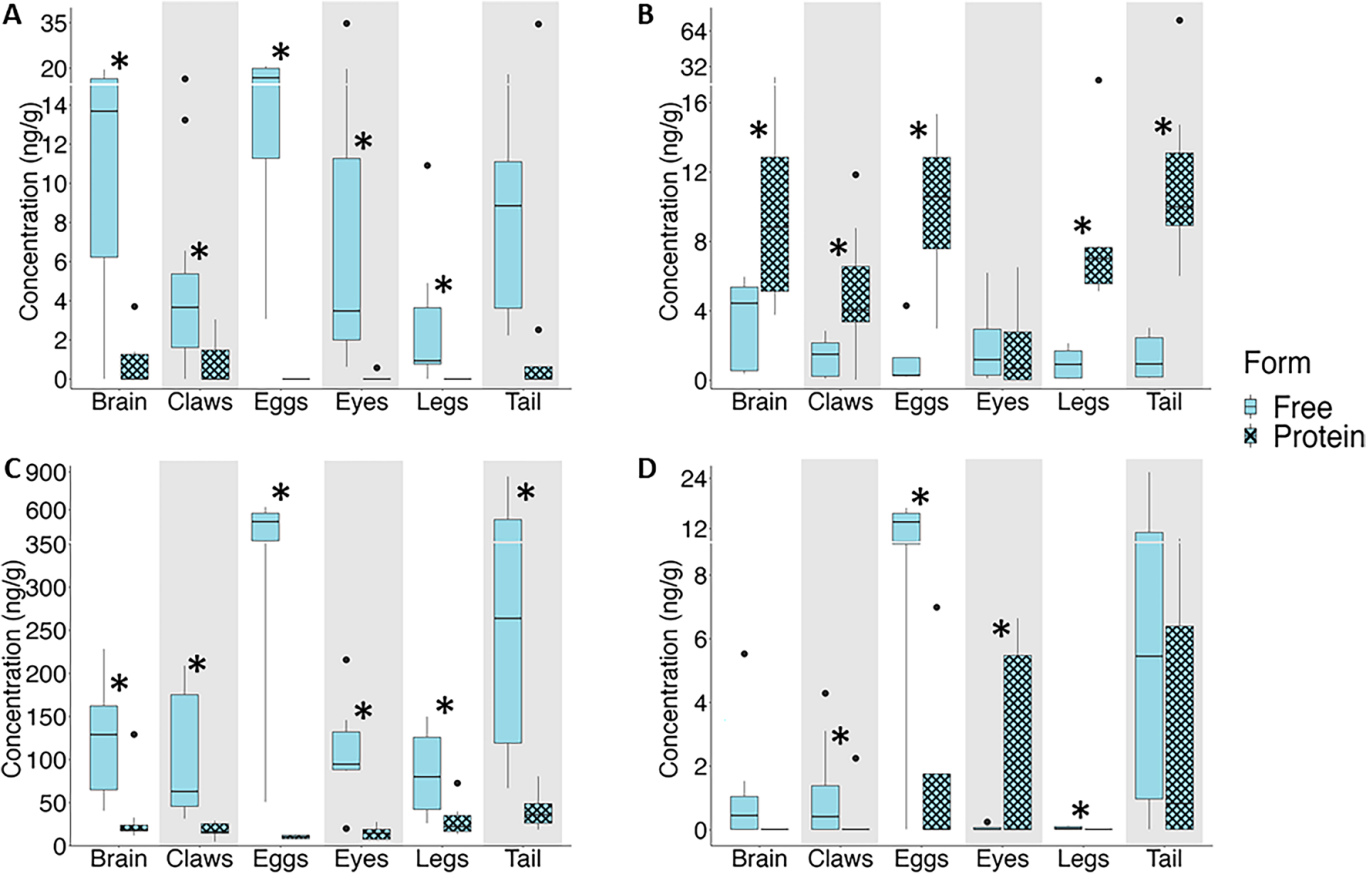
Concentration of non-protein amino acids in excised tissues of American Lobster (*Homarus americanus*). (**A**) β-methylamino-L-alanine (BMAA); (**B**) N-(2-aminoethyl)glycine (AEG); (**C**) 2,4-diaminobutyric acid (DAB); (**D**) β-aminomethyl-L-alanine (BAMA). Amino acids detected in the trichloroacetic acid extraction are identified in solid bars. Amino acids detected in precipitated proteins are identified in cross hatch bars. Non-detected values were replaced with 1/5th the limit of detection for statistical analysis. Asterisk denotes significant statistical differences (student’s t-test) between free and protein-bound fractions at *p* < 0.05 (*n* = 8 except claws (*n* = 15) and eggs (*n* = 4)).

**Figure 3:**
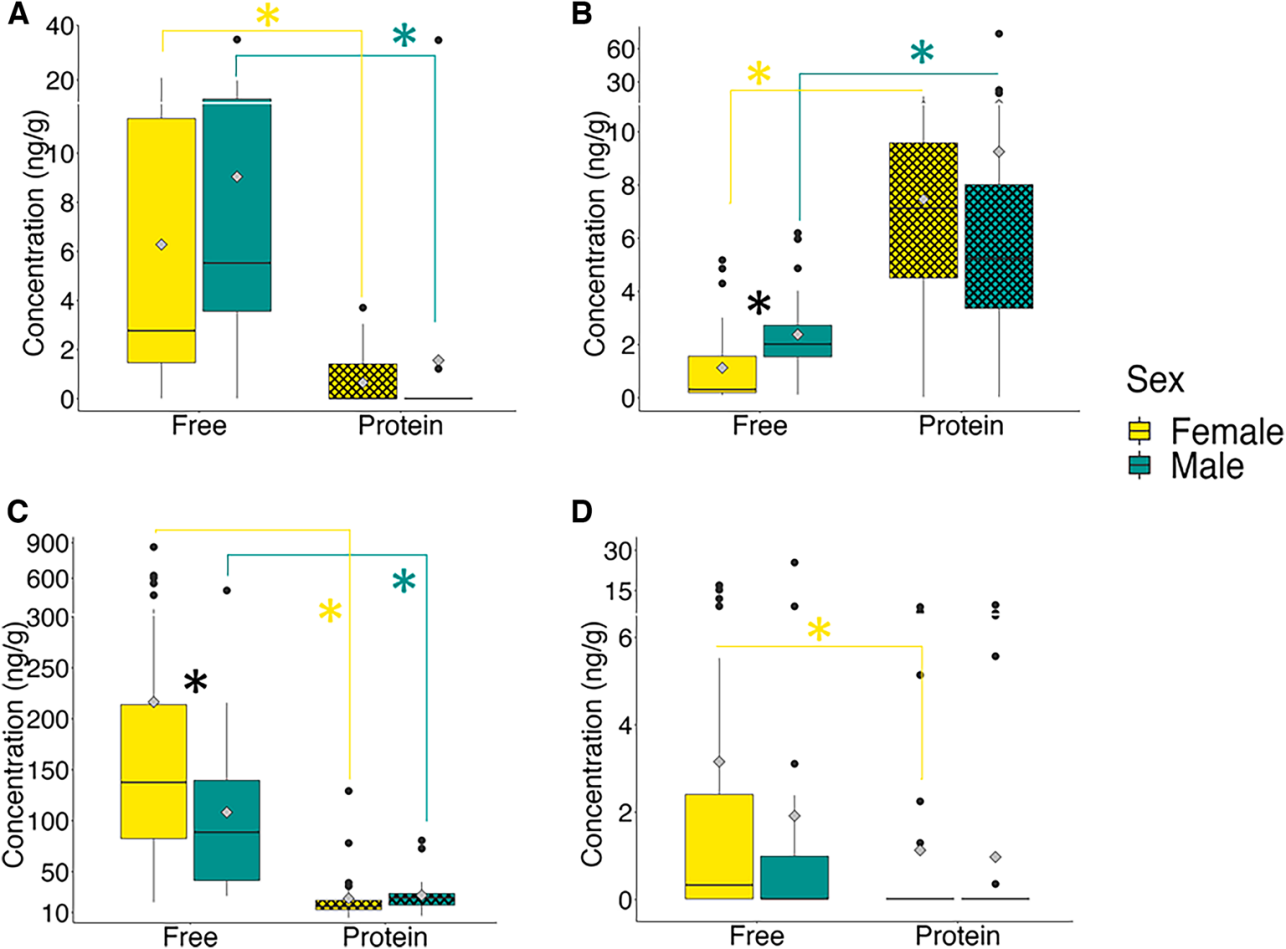
Concentration (ng/g) of non-protein amino acids in female and male American lobster (*Homarus americanus)* collected over two harvest seasons: (**A**) β-N-Methylamino-L-alanine (BMAA), (**B**) N-(2-aminoethyl) glycine (AEG), (**C**) 2,4-diaminobutyric acid (DAB) and (**D**) β-aminomethyl-L-alanine (BAMA). The sex is represented by color: yellow for females and green for males. The amino acids detected in the trichloroacetic acid extraction and the precipitated protein pellets are represented by solid and crosshatches respectively. The diamond within each boxplot shows the mean values. The non-detected values were substituted with 1/5^th^ the limit of detection for statistical analysis. Asterisk denotes significant differences (Student’s t-test) between sexes or different fractions within each sex (color coded) at *p* < 0.05 (*n* = 23 for male, *n* = 28 for female).

**Figure 4:**
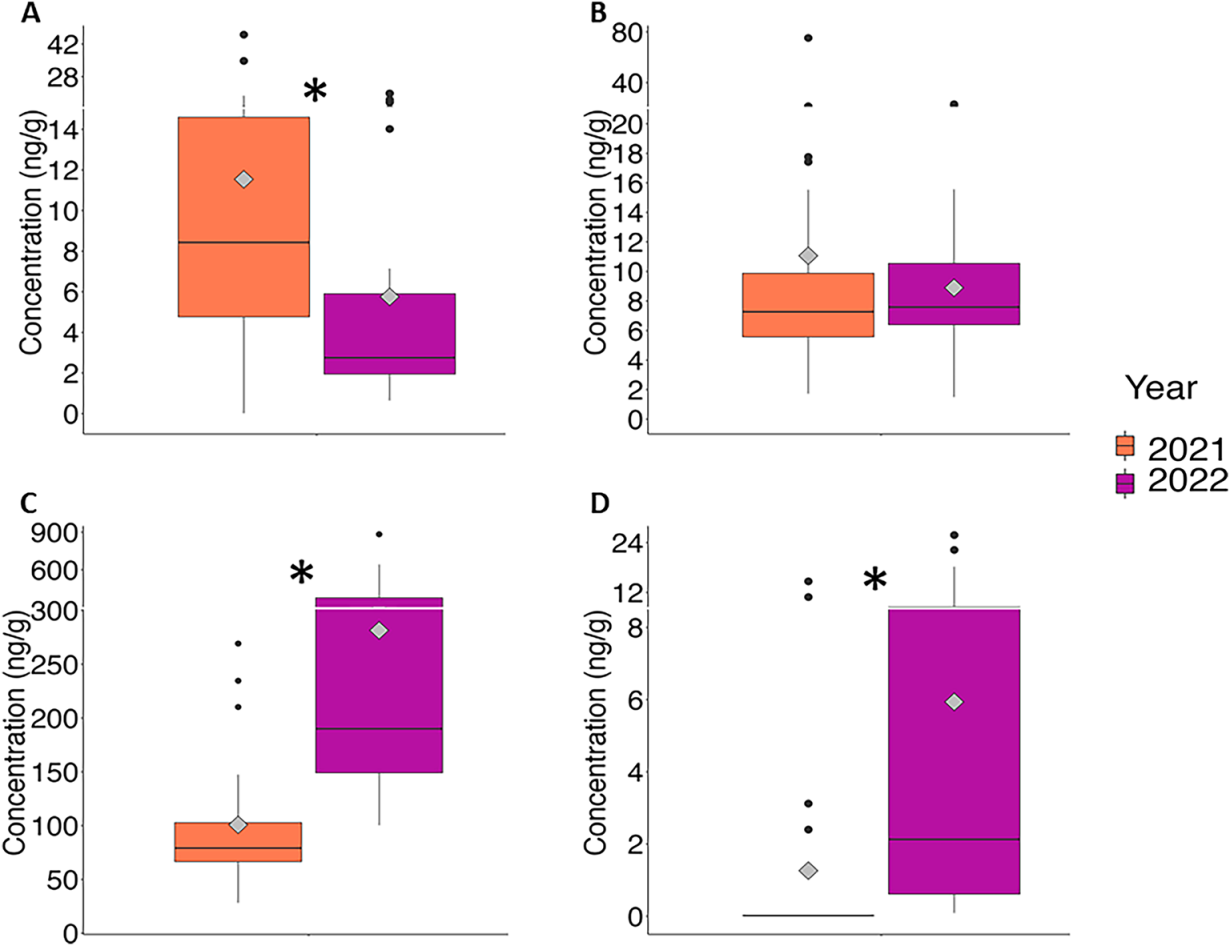
Concentration (ng/g) of non-protein amino acids across all tissues of American lobster (*Homarus americanus*) by harvest year. (**A**) β-methylamino-L-alanine (BMAA); (**B**) N-(2-aminoethyl)glycine (AEG); (**C**) 2,4-diaminobutyric acid (DAB); (**D**) β-aminomethyl-L-alanine (BAMA). The diamond within each boxplot shows the mean abundance. The non-detected values were substituted with 1/5th the limit of detection for statistical analysis. Asterisk denotes significant statistical differences (student’s t-test) at *p* < 0.05 (*n* = 25 for 2021 and *n* = 26 for 2022).

**Figure 5:**
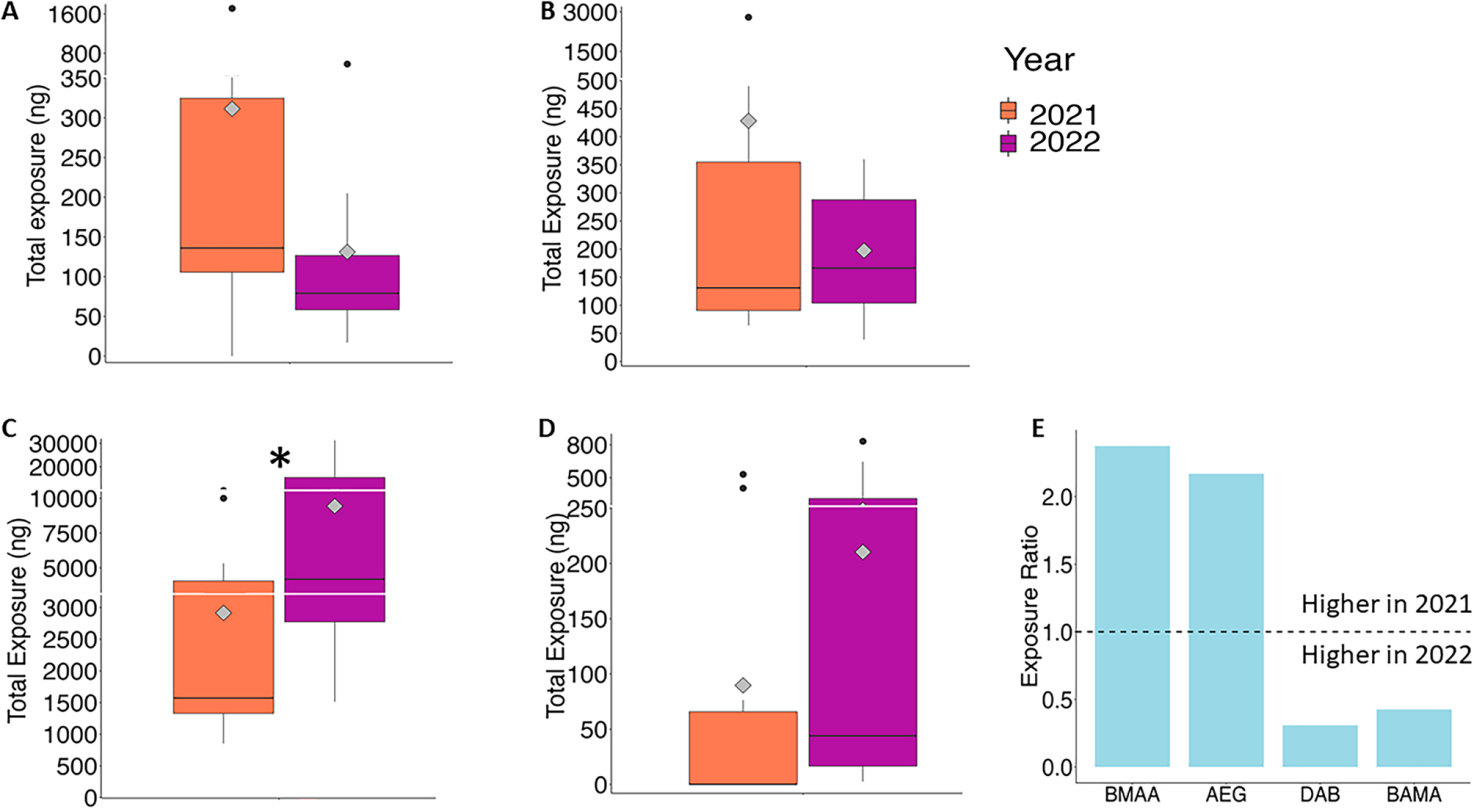
Estimated human exposure to non-protein amino acids based on consumption of claws and tails of American lobster (*Homarus americanus*) by year. (**A**) β-methylamino-L-alanine (BMAA); (**B**) N-(2-aminoethyl)glycine (AEG); (**C**) 2,4-diaminobutyric acid (DAB); (**D**) β-aminomethyl-L-alanine (BAMA). The diamond within each boxplot shows the mean abundance. The non-detected values were substituted with 1/5th the limit of detection. (**E**) Ratio for average exposure between the harvest 2021 vs 2022 for each of the NPAA (BMAA; AEG; DAB and BAMA). Ratios > 1 means exposure was higher in 2021 and ratios < 1 means exposure was higher in 2022. Asterisk denotes significant statistical differences (student’s t-test) at *p* < 0.05 (*n* = 8).

BMAA was detected in the brains, claws, eyes, eggs, legs and tails of the lobsters (Fig. 2A) with total concentration of up to 46.1 ng/g (Table S1). There was more BMAA detected in the free amino acid extracts than in the protein pellets. Proteins in the eyes, eggs and legs did not have detectable amounts of BMAA (Fig. 2A). AEG was also detected in the brains, claws, eggs, eyes, legs and tails of the lobsters (Fig. 2B) with total concentration higher than BMAA. AEG was also significantly higher in the protein pellet than in the free amino acid extractions of all the tissues except eyes (Fig. 2B). DAB was the most abundant NPAA with concentrations of up to 883.4ng/g and was detected in all tissues. There was significantly more DAB in the free amino acid extractions than in the protein pellets (Fig. 2C). The distribution of BAMA was slightly different than the other NPAAs with significant amounts of BAMA in the lobster tails, BAMA in the protein pellets of tails, eggs and eyes and BAMA in the free amino acid extractions of brain, claws, eggs and tails (Fig. 2D). We investigated whether male and female lobsters are metabolizing NPAAs differently (Fig. 3A–D). There were no significant differences in the protein-pellets of all NPAAs or free amino acid extracts for BMAA and BAMA (Fig. 3A–D). However, AEG was higher in the free amino acid extracts of male lobsters (Fig. 3B) while DAB was higher in the free amino acid extracts of female lobsters (Fig. 3C). We found a significant difference in the amounts of NPAAs in the two harvest years. The abundance of BMAA was approximately three-times higher in the harvest 2021 compared to 2022 (Fig. 4A). The abundance of AEG did not differ between years (Fig. 4B) while the abundance of DAB was ~ 13 times higher in harvest 2022 than in harvest 2021 (Fig. 4C). BAMA was also higher in harvest 2022 (Fig. 4D) and was detected in only four samples (tail and claws of two lobsters) in 2021 (Table S1). We estimated the human exposure to BMAA and the other isomers if only the most popular tissues—claws and tails—were eaten (Fig. 5). A consumer eating on the claws and tails of lobsters caught in 2021 would be exposed to approximately twice the amount of BMAA as the same meal consumed in 2022 while exposure to DAB and BAMA would higher from harvest of 2022 (Fig. 5).

## Discussion

Our study was prompted by recent events in the Province of New Brunswick on the Atlantic coast of Canada. Since 2015, the Office of the Chief Medical Officer of Health for New Brunswick has issued public advisories for cyanobacterial blooms for 23 water bodies (https://www2.gnb.ca/content/gnb/en/departments/ocmoh/health_advisories.html). At the same time, a cluster of neurological disease has been reported to the Public Health Agency of Canada (PHAC) and investigated by the Public Health New Brunswick (PHNB)^46^. The Oversight Committee reported that between the fall of 2020 and the end of April 2021, a total of 48 individuals had been formally identified as neurological patients in this cluster^46^. Medical records, patient records, a survey of environmental and dietary exposures^47^ and 6 autopsy reports were assessed^46^. Ultimately, the PHNB determined “*the individuals who were included in this cluster do not represent a neurological syndrome of unknown cause and has therefore concluded that no such syndrome exists. No individual met the case definition in full and many were found to have other, more probable diagnoses*”^46^. While the specific criteria of a syndrome of unknown cause was not met, ongoing studies continue to assess the risks of neurological ailments in the region. One of the interesting pieces of data in the report concerns environmental and dietary exposures^46,47^. Thirty-four of the 48 patients participated in this study and 31 (91.2%) of the patients reported eating fresh or frozen lobster in the 2 years prior to the onset of symptoms^47^. American lobster spends most of its life in shallow waters and is harvested close to shore depths less than 40 m in Canada^48^ and thus are exposed to changes occurring on the surface and lower depths of oceans such as harmful algal blooms. Our objective was to quantify the human exposure to BMAA and its isomers through the consumption of lobsters harvested in Atlantic Canada and distributed across the country in 2021 and 2022.

BMAA has previously been reported in lobster harvested from northwest Atlantic^20,21^, as well as other seafood ^17–19^ and aquatic animals^22–25^. The association between cyanobacterial blooms and contamination of seafood with BMAA is well established^13,26,49^. BMAA was present at lower concentrations in protein pellets as compared to free amino acid extracts of lobster tissues which is consistent with previous reports^21^ and may indicate that lobsters may avoid incorporation of BMAA into their proteins^2^ and potentially excreting rather than accumulating BMAA in tissues. Our data indicate that the concentrations of BMAA could vary between harvests. The observed concentrations of BMAA are also lower than the concentrations reported to cause acute toxicity but previous research has demonstrated neurological abnormalities from prolonged exposure to low concentrations of BMAA in vervets^34,35^ and human neural models^50–52^.

BMAA can cause cellular defects such as protein misfolding, neuroinflammation, disruption in signaling pathways, and other mechanisms that may result in neurodegeneration with chronic exposure^51–53^. Several recent reviews have investigated the prevalence of BMAA in ecosystems and potential neurotoxicity^54,55^. Less is known about the ecological impacts or neurotoxicity of the other NPAAs. We also found higher amounts of DAB in the free amino acid extracts than in the protein pellet. In Zebrafish larva, DAB was found to be a more potent neurotoxin than BMAA^56^. Interestingly, AEG, a gamma amino acid, was found at higher concentrations in protein pellet than the free amino acid extracts. AEG modulated startle kinetics and modified neurological responses in the Zebrafish model of neurotoxicity^56^. In evolution, AEG formed part of the peptide nucleic acid polymer that predated RNA^57^ and we have previously reported isolation and identification of AEG from ancient microbialites collected from Pavilion Lake, British Columbia^15^. These data demonstrate that AEG could also be bioaccumulated in lobsters. Finally, this study detected BAMA in lobster tissues which is significant because it has previously only been quantified in cyanobacterial blooms and water samples and its bioactivity is unknown^15,16,58,59^. An interesting observation is that BAMA was detected in the tail and both claws of one male lobster and the tail of a second male lobster caught in 2021. In the 2022 harvest, BAMA was detected and quantified in all 4 lobsters and 25/26 lobster samples (see Table S3). Further research is needed to understand the metabolism, bioactivity and ecotoxicology of BAMA.

Overall, our data indicate that cyanobacterial NPAA neurotoxins are accumulated in lobsters. The amounts of detectable NPAAs varied by harvest year, tissue, and sex of the animals. The human exposure to these neurotoxins can be different from year to year, depending on environmental conditions. Recommendations for regular testing protocols to determine risks could support the lobster industry in Canada and elsewhere.

## Supplementary Information

Supplementary Information.

## Data Availability

All data generated or analyzed during this study are included in this published article [and its supplementary information files].
