# Author Correction: Neurotoxic non-protein amino acids in commercially harvested Lobsters (*Homarus americanus* H. Milne-Edwards)

**DOI:** 10.1038/s41598-025-00860-3

**Published:** 2025-05-19

**Authors:** Pawanjit K. Sandhu, Julia T. Solonenka, Susan J. Murch

**Affiliations:** Department of Chemistry, University of British Columbia, Syilx Okanagan Nation Territory, Kelowna, BC V1V 1V7 Canada

Correction to: *Scientific* *Reports* 10.1038/s41598-024-58778-1 Published Online 05 April 2024.

The original version of this Article contained errors.

Statements referring to correlations between lobster BMAA levels detected in the study and cyanobacterial activity in the region were not supported by data. Additionally, the findings of the study had not been adequately contextualised with reference to previous literature on the effects of chronic exposure to low levels of BMAA.

In the Abstract,

"Our quantification data varied by individual lobster, sex and collection year. Significantly more BMAA was quantified in lobsters harvested in 2021 than 2022. Interestingly, more BAMA was quantified in lobsters harvested in 2022 than 2021. Monitoring of lobster harvests for cyanobacterial neurotoxins when harmful algal bloom events occur could mitigate risks to human health."

now reads:

"Our quantification data varied by individual lobster, sex and collection year. Significantly more BMAA was quantified in lobsters harvested in 2021 than 2022. Interestingly, more BAMA was quantified in lobsters harvested in 2022 than 2021. The concentrations of BMAA we observed in lobsters are lower than an acutely toxic dose, but given previous research which has demonstrated that chronic exposure to low levels of BMAA can cause neurological abnormalities, we propose continued monitoring of lobster harvests for cyanobacterial neurotoxins to assess potential risks to human health."

Additionally, in the Discussion section,

"Our data indicate that the concentrations of BMAA could vary between years depending upon the activity of cyanobacterial blooms in the habitat. This is in line with our observation that BMAA concentration was lower in lobsters harvested in 2022, potentially coinciding with a reduction in the active cyanobacterial blooms in the region. The observed concentrations of BMAA are lower than the concentrations reported to cause acute toxicity^34,50,51^."

now reads:

"Our data indicate that the concentrations of BMAA could vary between harvests. The observed concentrations of BMAA are also lower than the concentrations reported to cause acute toxicity but previous research has demonstrated neurological abnormalities from prolonged exposure to low concentrations of BMAA in vervets^34,35^ and human neural models^50,51,52^."

The original Article has been corrected.

