# Supplementary material for: Neurotoxic non-protein amino acids in commercially harvested Lobsters (Homarus americanus H. Milne-Edwards)
Source: Sci Rep. 2024 Apr 5;14:8017. doi: 10.1038/s41598-024-58778-1 (PMC10997655; doi:10.1038/s41598-024-58778-1)
Supplement: Supplementary file 1 — Supplementary Information. [file 41598_2024_58778_MOESM1_ESM.docx]

Supplemental Information

Non-protein amino acids in commercially harvested Lobsters (*Homarus americanus* H. Milne-Edwards).

Pawanjit K Sandhu^1^, Julia T Solonenka^1^, Susan J Murch^1^*

^1^Department of Chemistry, University of British Columbia, Syilx Okanagan Nation Territory, Kelowna, BC – V1V 1V7

*Corresponding author: [susan.murch@ubc.ca](mailto:susan.murch@ubc.ca)

**Method Performance Characteristics**

**
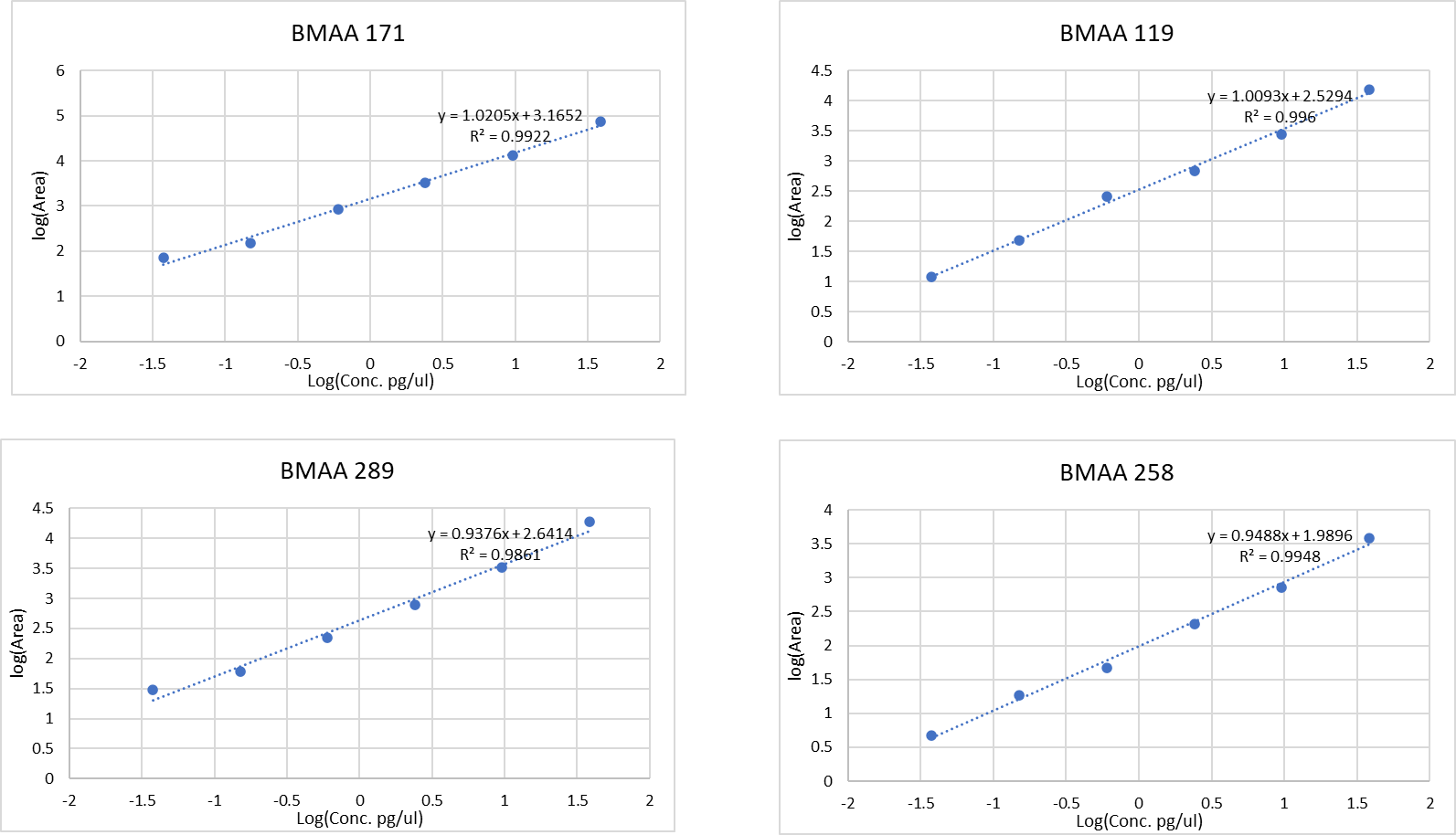
Figure S1**: Representative standard curves for the detection and quantification of non-protein amino acids: β-methylamino-L-alanine (BMAA); N-(2-aminoethyl)glycine (AEG); 2,4-diaminobutyric acid (DAB); β-aminomethyl-L-alanine (BAMA) in American lobster tissues.


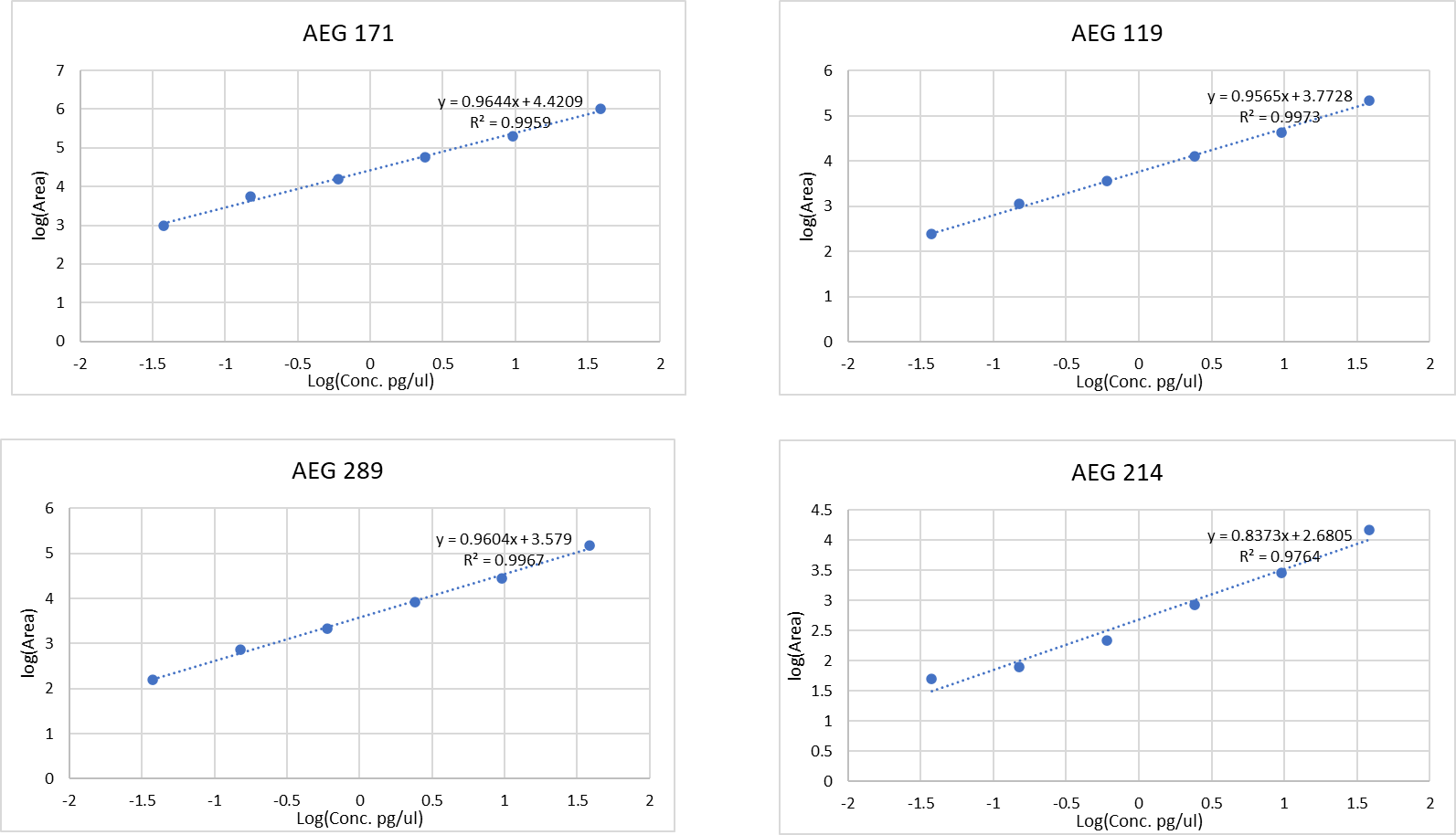


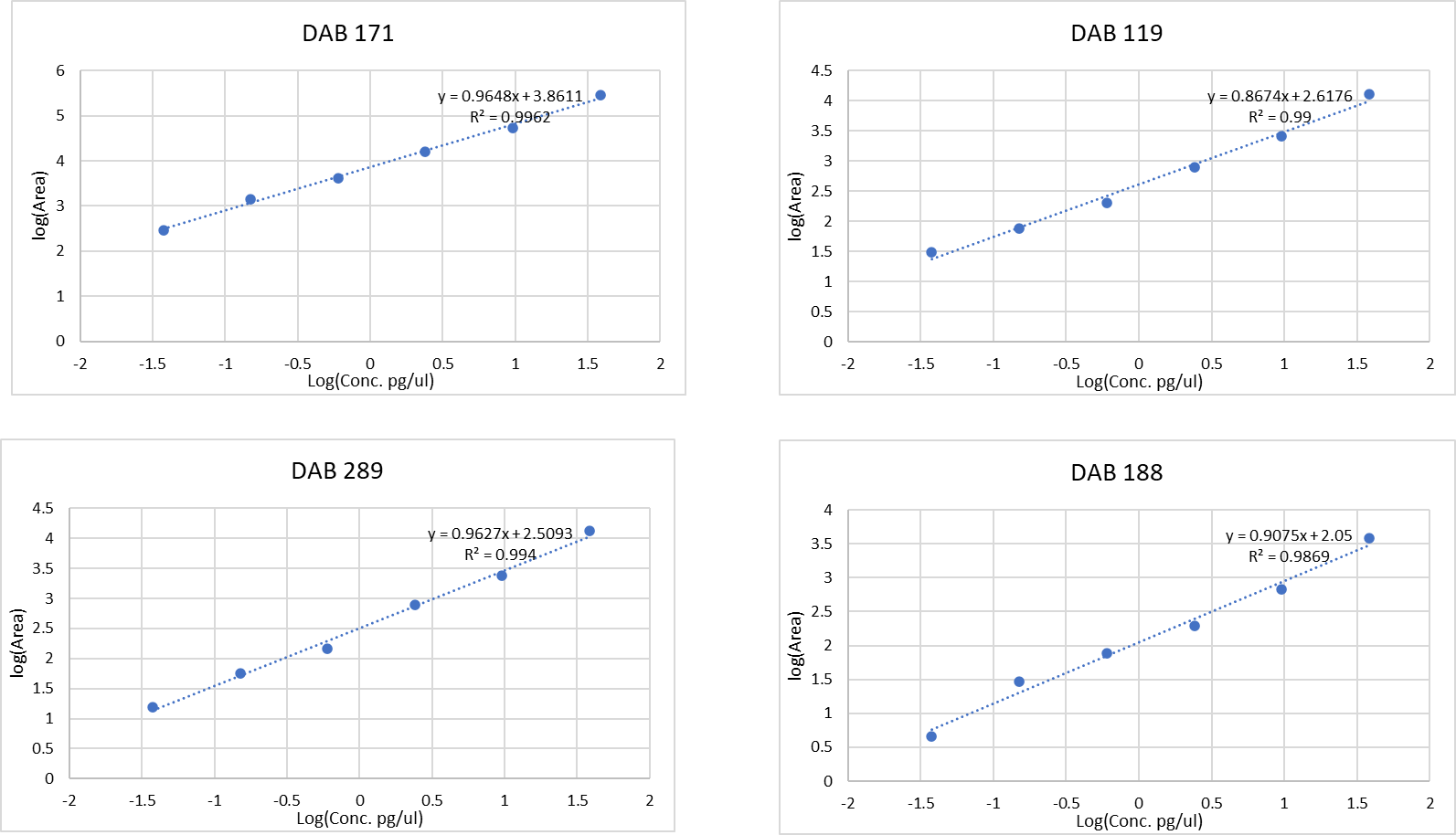


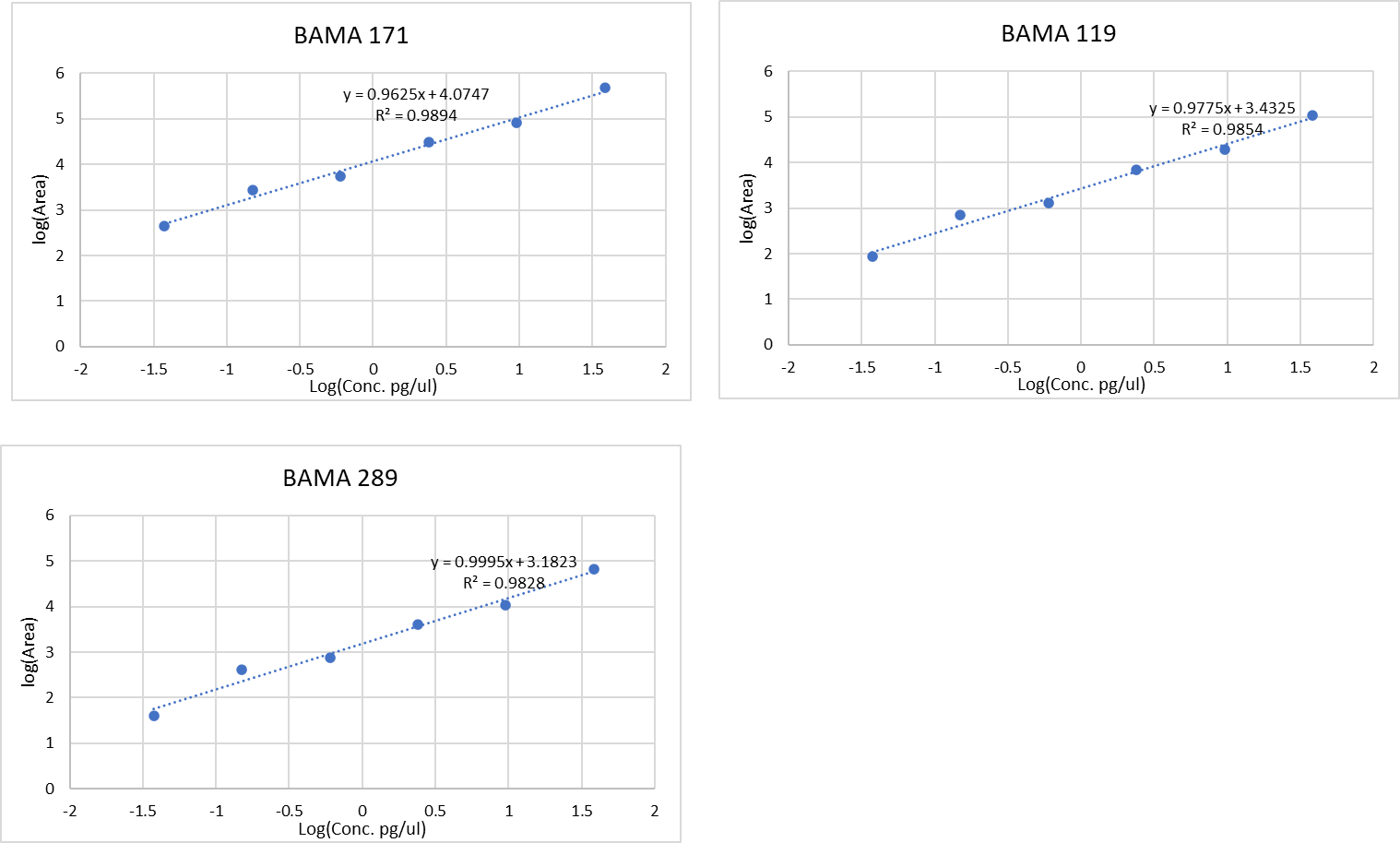


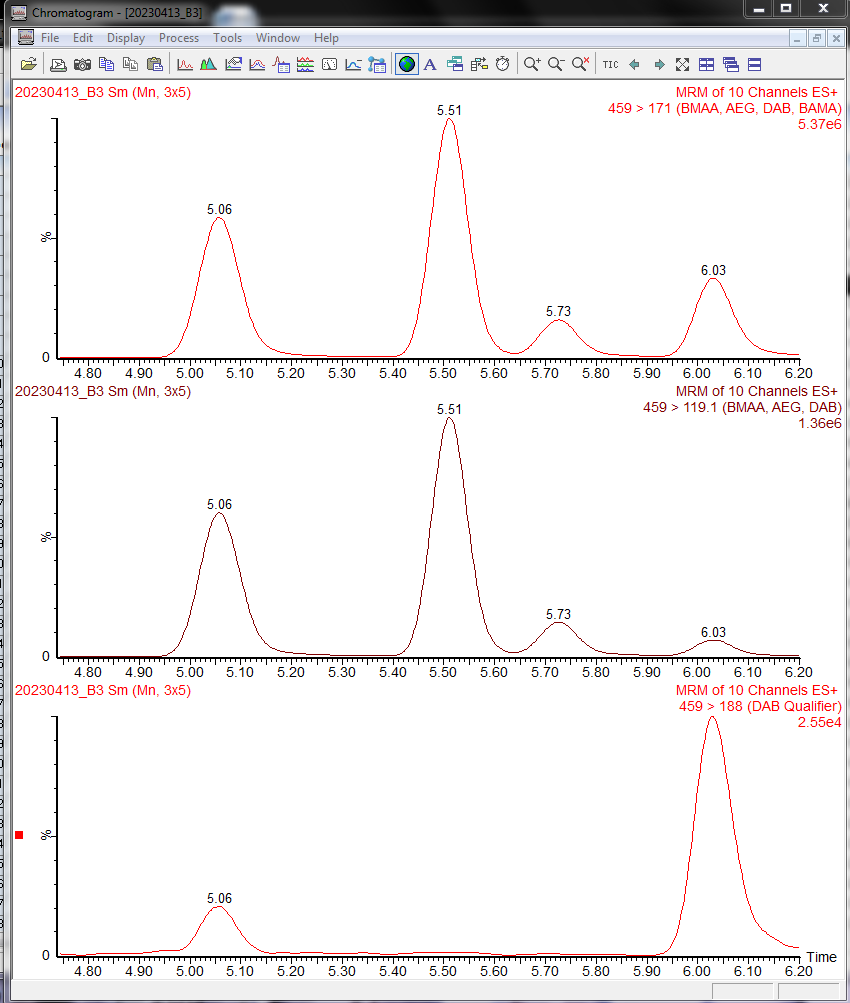

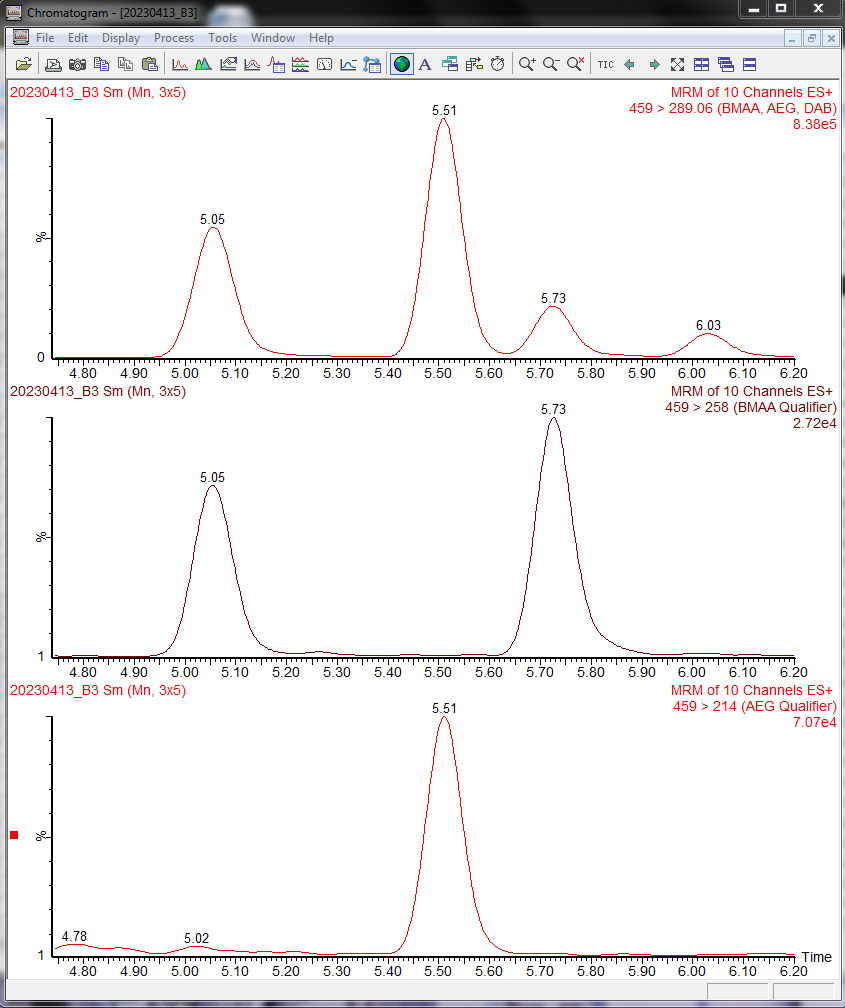

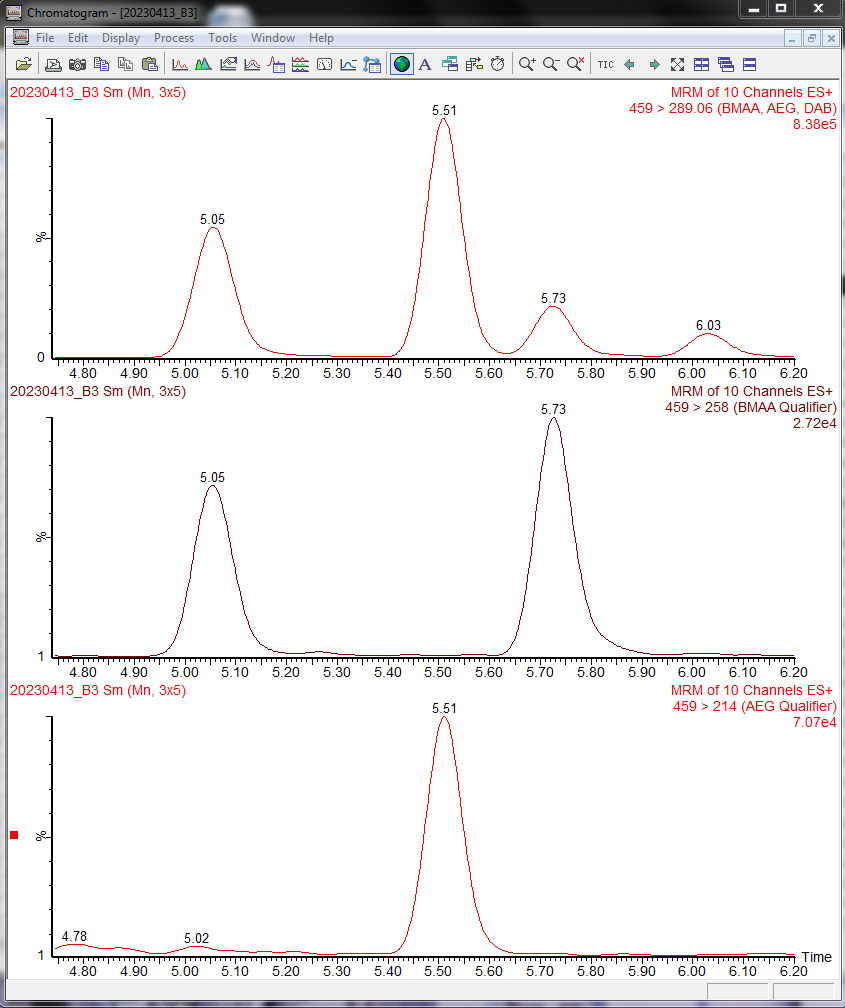

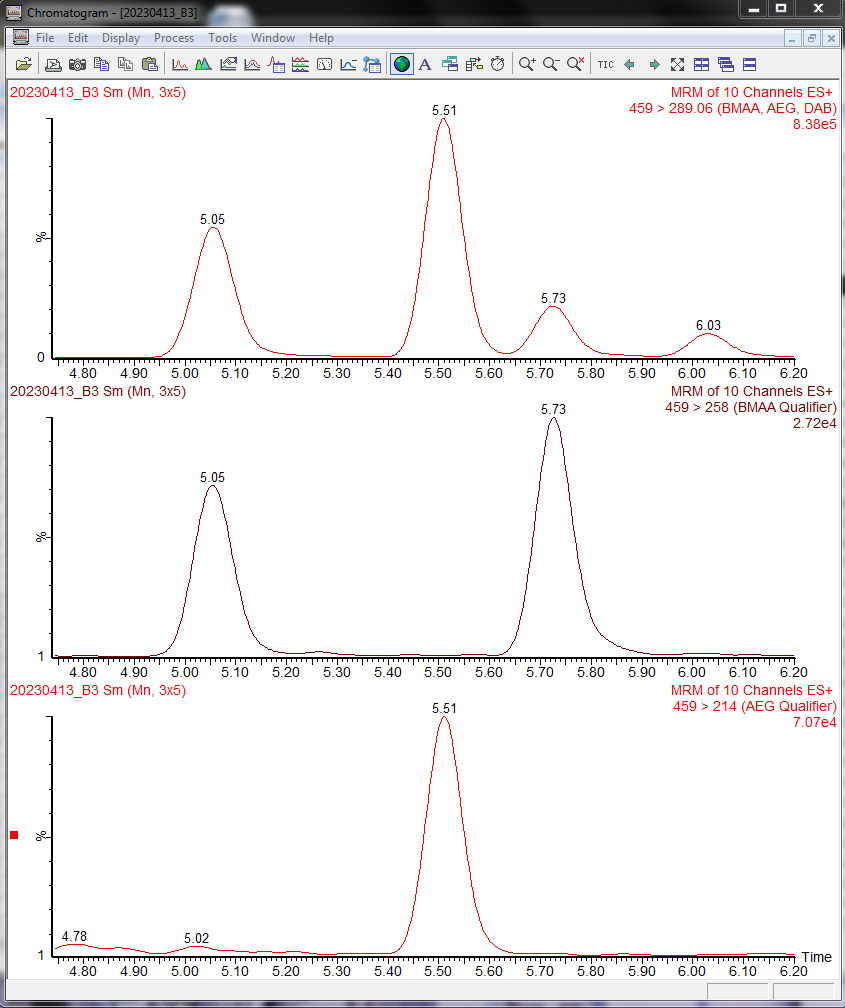

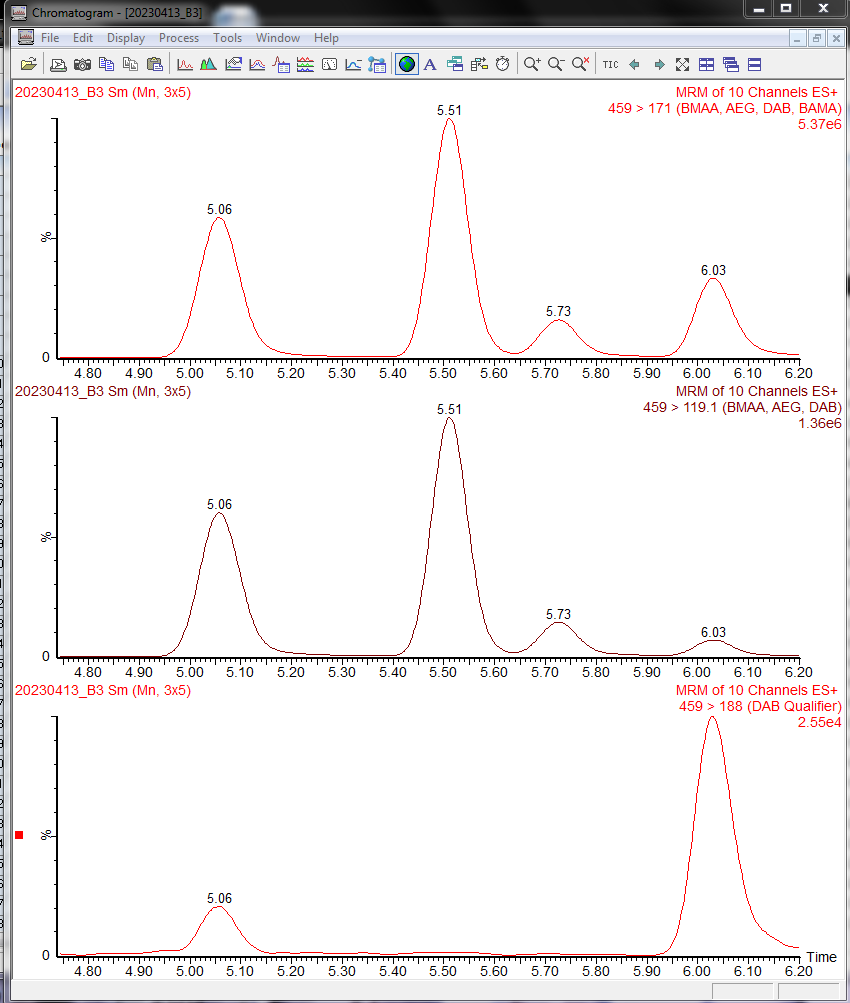

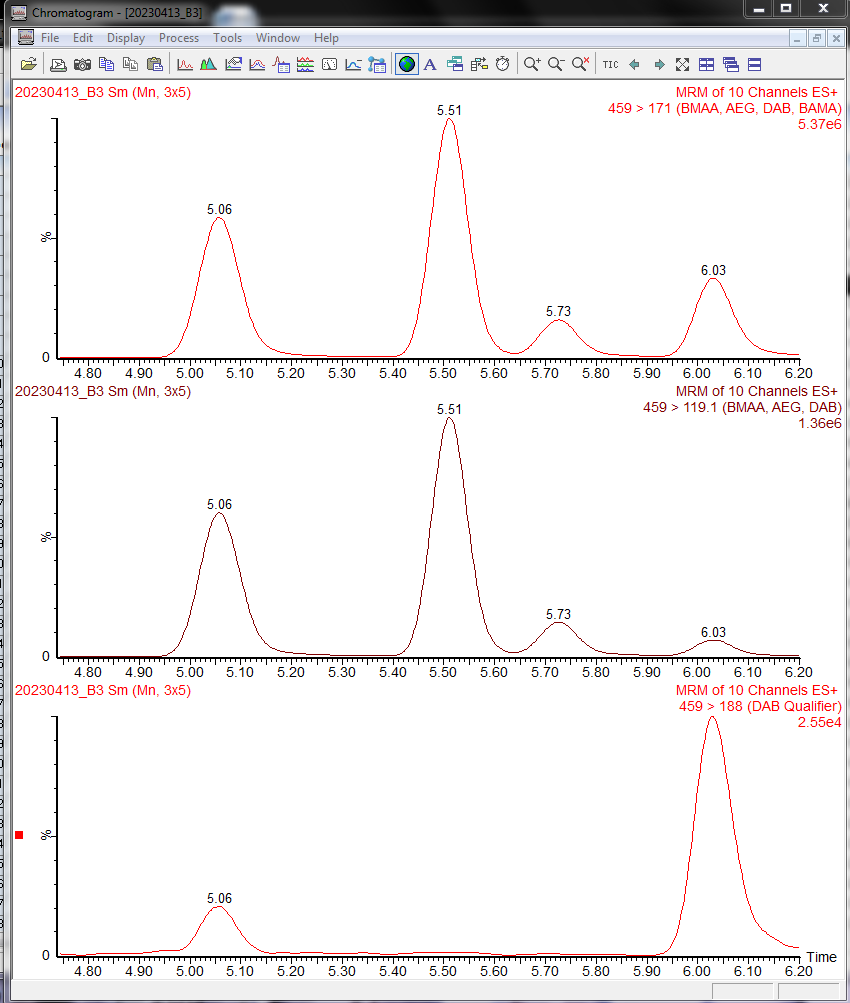
**Figure S2**: Chromatogram of a representative standard mix displaying quantifier and qualifier transitions for each of the NPAA. X-axis in each of the windows represents time and Y-axis relative intensity (RI). The order of elution is BAMA>AEG>BMAA>DAB.

RI

RI

RI

RI

RI

RI

Time

Time

Time

Time

Time

Time

Quantifier 459 >171

Qualifier 459 >119.1

Qualifier 459 >289.06

BMAA Qualifier 459 >258

AEG Qualifier 459 >214

DAB Qualifier 459 >188


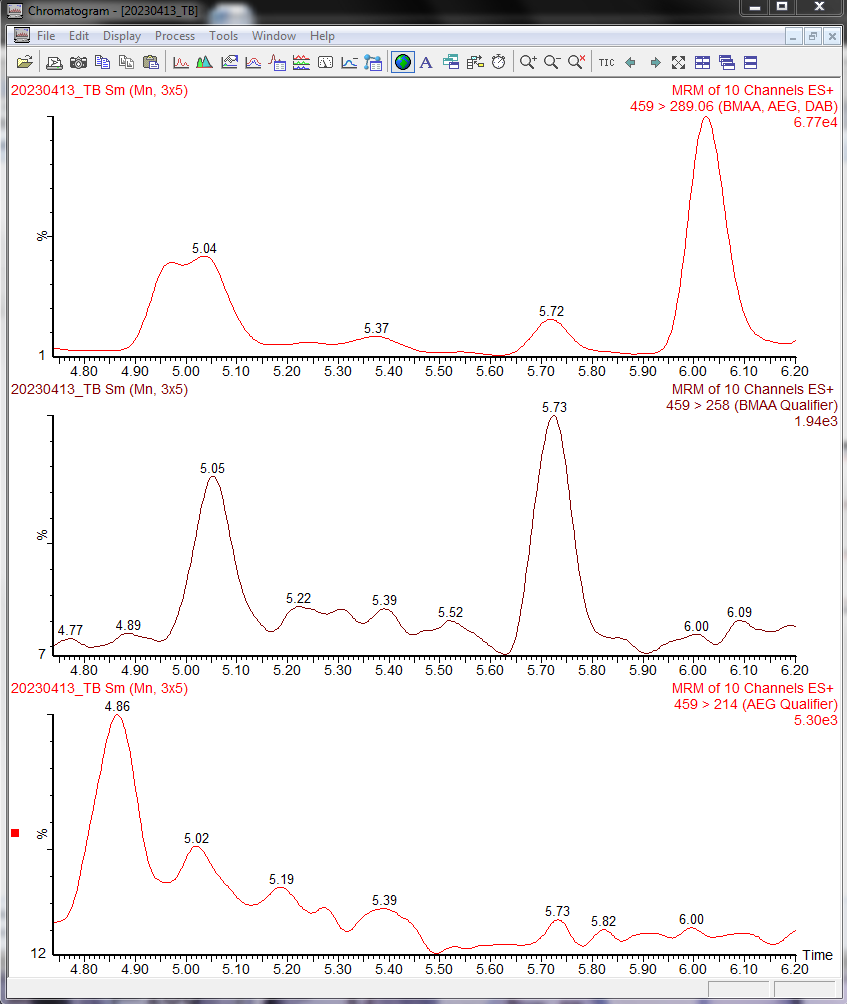

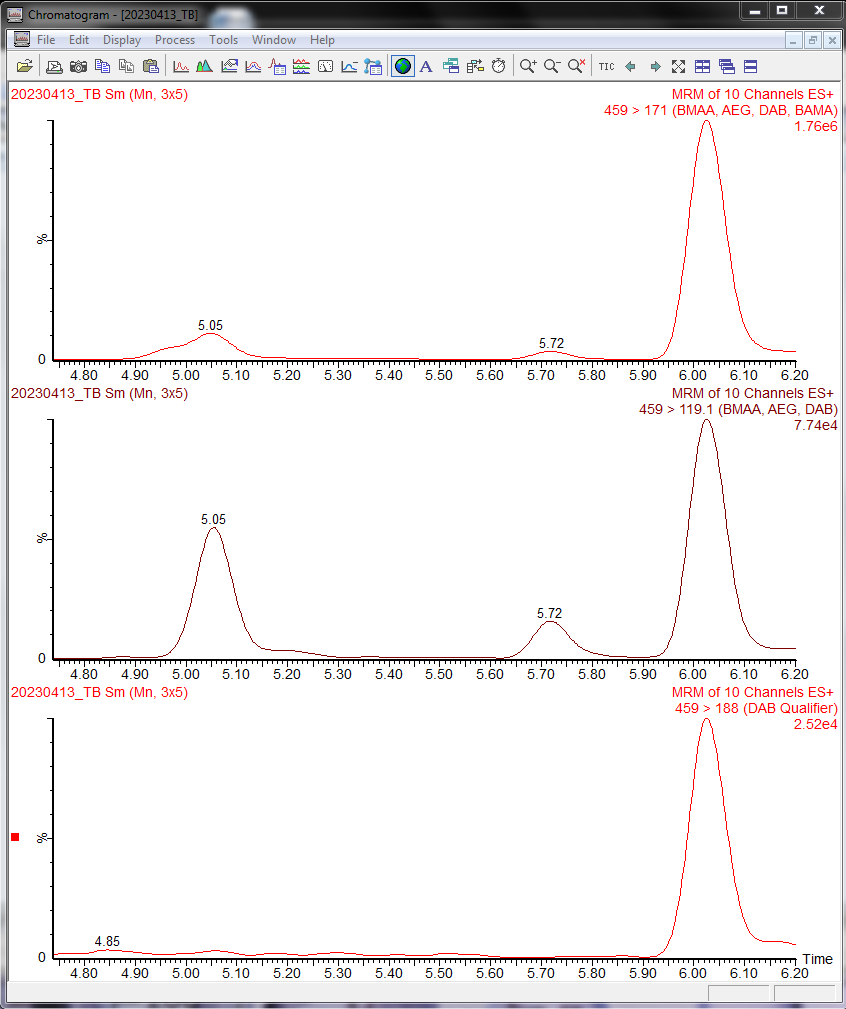

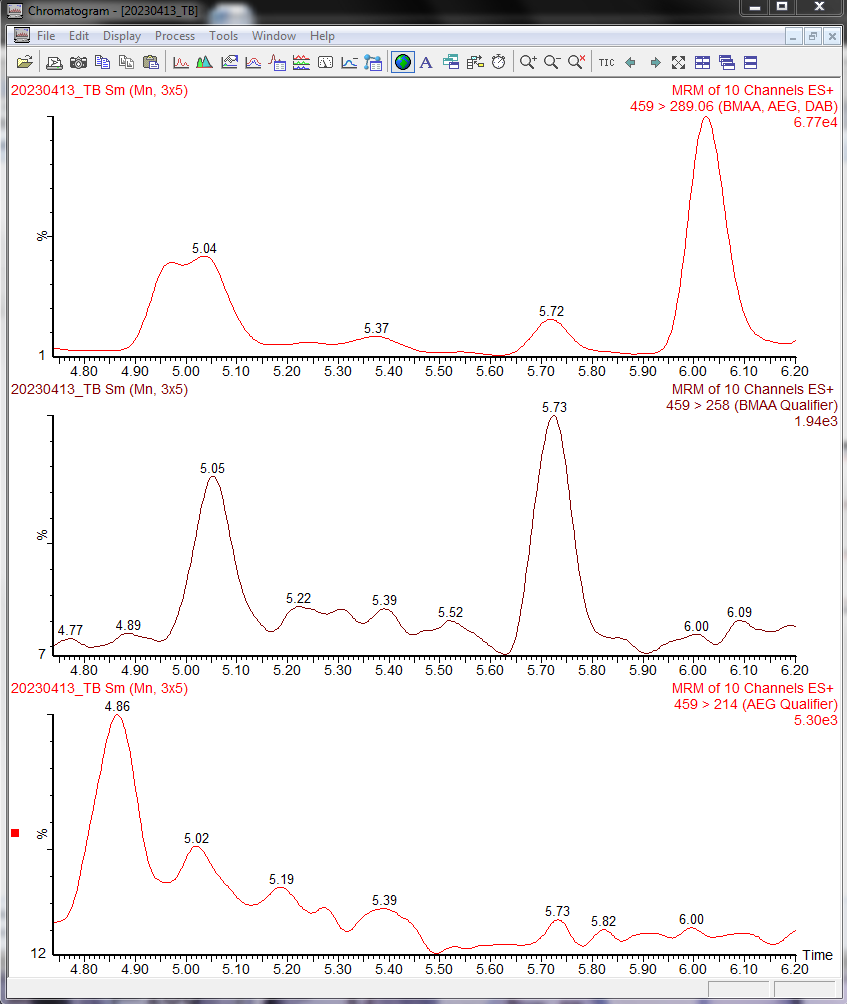

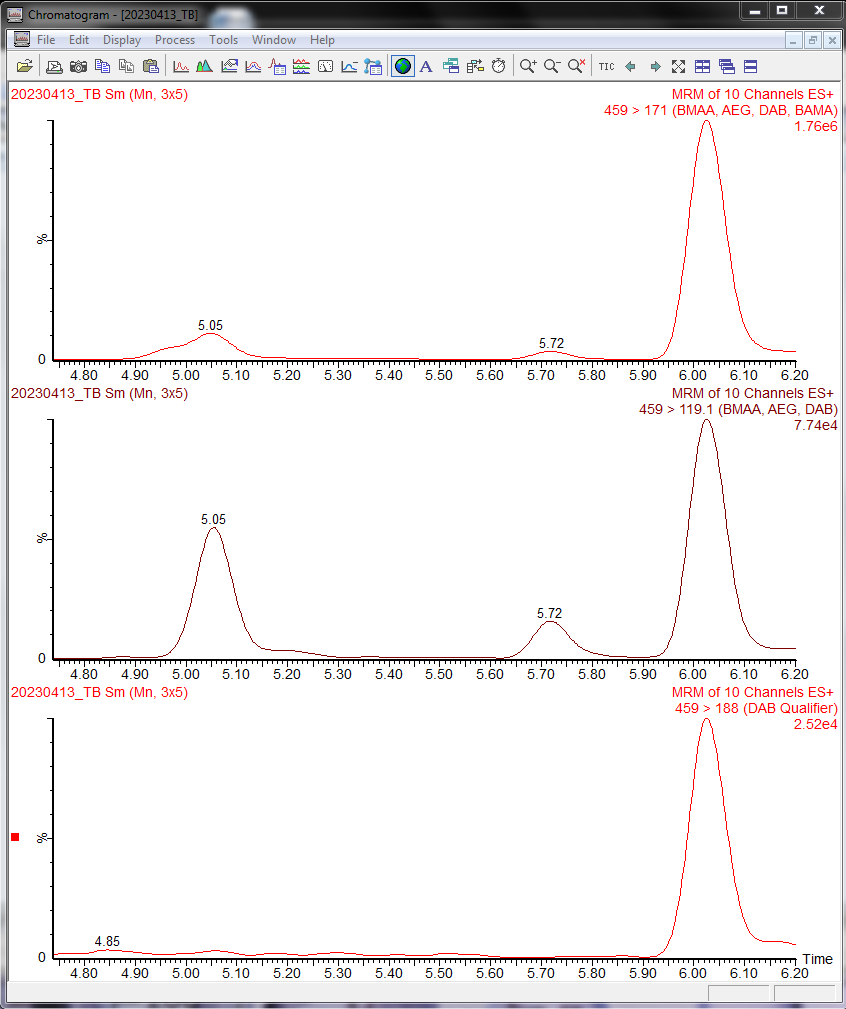

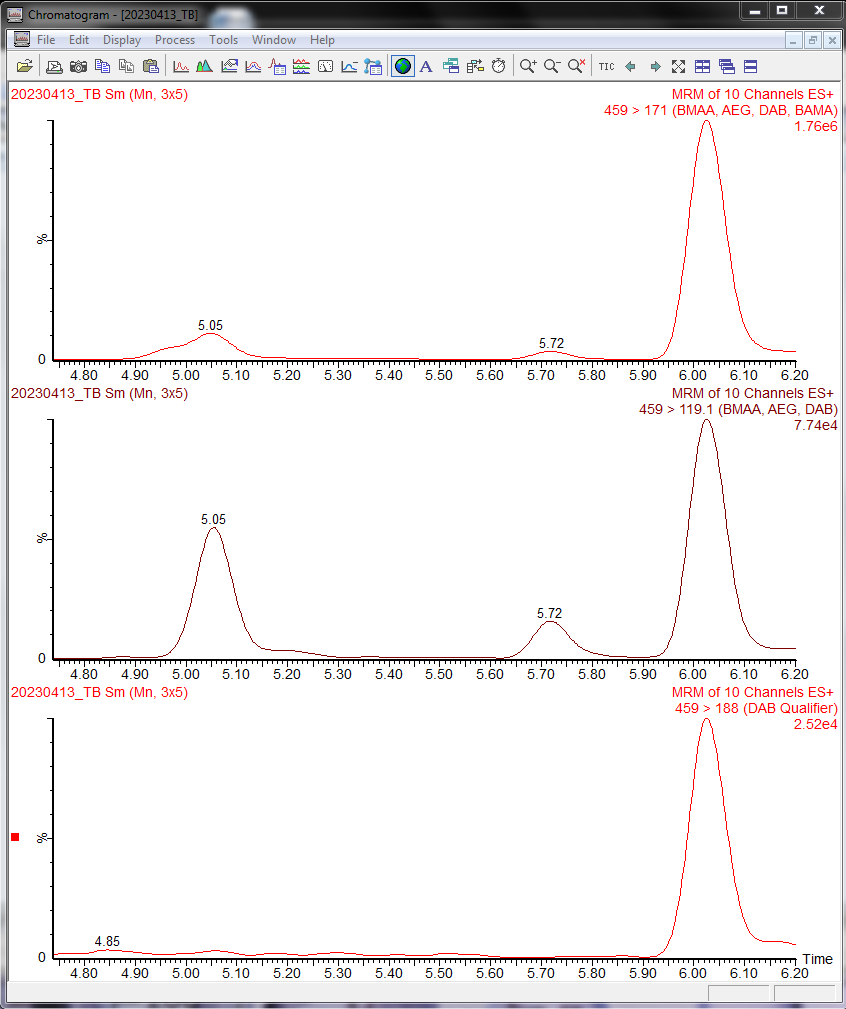

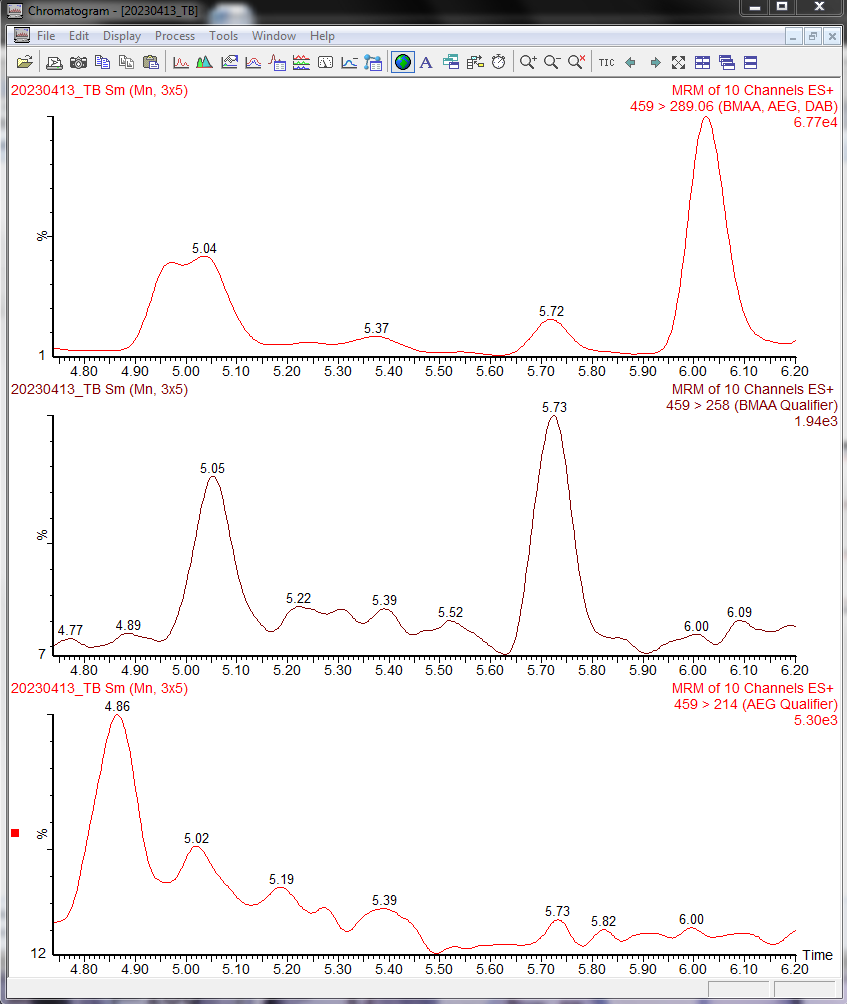
**Figure S3**: Chromatogram of a representative lobster sample displaying quantifier and qualifier transitions. The sample had BAMA, BMAA and DAB above the instrumental detectable limits while AEG was undetectable. X-axis in each of the windows represents time and Y-axis relative intensity (RI). The order of elution is BAMA>AEG>BMAA>DAB.

Quantifier 459 >171

Qualifier 459 >119.1

Qualifier 459 >289.06

BMAA Qualifier 459 >258

AEG Qualifier 459 >214

DAB Qualifier 459 >188

Time

Time

Time

Time

Time

Time

RI

RI

RI

RI

RI

RI

**Table S1**: Percentage Relative Standard Deviation (RSD) in signal response.

| % RSD (Inter-day variability) | | | | |
| --- | --- | --- | --- | --- |
| Standard | BMAA | AEG | DAB | BAMA |
| S7 | 64.8 | 9.7 | 45.2 | 32.7 |
| S6 | 10.7 | 14.5 | 15.8 | 10.7 |
| S5 | 18.1 | 17.5 | 11.3 | 17.4 |
| S4 | 3.1 | 2.9 | 8.0 | 9.1 |
| S3 | 7.6 | 11.2 | 21.4 | 9.7 |
| S2 | 7.8 | 9.6 | 21.8 | 2.8 |
| Average | 18.7 | 10.9 | 20.6 | 13.7 |

| Compound | 0.1N TCA extract | Hydrolysed protein pellet |
| --- | --- | --- |
| BMAA | 109.3 | 28.3 |
| AEG | 48.3 | 34.2 |
| DAB | 81.2 | 24.8 |
| BAMA | 78.8 | 13.9 |

**Table S2**: Average spike recovery (%) of BMAA, AEG, DAB and BAMA from 0.1N trichloroacetic acid extract and 6N HCl hydrolyzed protein pellet from different tissues of American lobster.

**Table S3:** Concentration (ng/g tissue weight) of free and protein-bound BMAA, AEG, DAB and BAMA in tissues of American lobster. Free represents value from TCA extraction and protein-bound represents values from precipitated proteins. L1-L4 are lobsters from harvest 2021 and L5-L8 are lobsters from harvest 2022. ND represents non-detected values, D that were detected but were below lower limit of quantification (LLOQ). ND values were replaced with one-fifth the Limit of Detection (LOD) for statistical analysis.

| Year | Lobster | Sex | Organ/  Tissue | Free (ng/g) | | | | Protein-bound (ng/g) | | | |
| --- | --- | --- | --- | --- | --- | --- | --- | --- | --- | --- | --- |
|  |  |  |  | **BMAA** | **DAB** | **AEG** | **BAMA** | **BMAA** | **DAB** | **AEG** | **BAMA** |
| 2021 | L1 | Female | Eyeballs | 4.44 | 20.08 | 1.71 | ND | ND | 8.04 | ND | ND |
|  |  |  | Legs | 10.91 | 45.21 | D | ND | ND | 33.64 | 7.63 | ND |
|  |  |  | Tail | 10.96 | 66.88 | 3.02 | ND | ND | 35.92 | 14.74 | ND |
|  |  |  | Brain | 12.78 | 69.05 | 4.86 | ND | ND | 18.88 | 4.8 | ND |
|  |  |  | Claw | 13.23 | 56.36 | 2.77 | ND | ND | 10.23 | ND | ND |
|  |  |  | Claw | 16.49 | 49.95 | 2.85 | ND | ND | 29.18 | D | ND |
|  |  |  | Eggs | 19.71 | 50.8 | 4.3 | ND | ND | 12.15 | D | ND |
| 2021 | L2 | Male | Eyeballs | 19.82 | 215.8 | 6.2 | ND | ND | 18.81 | ND | ND |
|  |  |  | Legs | 4.91 | 66.53 | D | ND | ND | 25.76 | D | ND |
|  |  |  | Tail | 11.54 | 188.5 | D | D | 34.54 | 80.76 | 73.55 | 9.63 |
|  |  |  | Brain | 7.38 | 52.82 | 5.97 | ND | ND | 18.02 | 11.45 | ND |
|  |  |  | Claw | 4.36 | 42.44 | D | ND | ND | 24.4 | 4.05 | ND |
|  |  |  | Claw | 6.56 | 63.17 | D | ND | ND | 25.62 | D | ND |
| 2021 | L3 | Male | Eyeballs | 34.76 | 96.46 | 4.87 | ND | ND | 6.71 | ND | ND |
|  |  |  | Legs | 3.23 | 33.27 | D | ND | ND | 40.08 | 7.74 | ND |
|  |  |  | Tail | 10.57 | 111.4 | 2.98 | 9.13 | ND | 36.05 | 12.56 | 5.57 |
|  |  |  | Brain | 14.59 | 40.6 | 4.03 | ND | ND | 21.05 | D | ND |
|  |  |  | Claw | 5.53 | 49.28 | 2.33 | 3.11 | ND | 25.89 | D | ND |
|  |  |  | Claw | 5.23 | 32.32 | D | 2.39 | ND | 29.43 | D | ND |
| 2021 | L4 | Male | Eyeballs | 8.42 | 93.08 | 2.31 | ND | ND | 6.88 | ND | ND |
|  |  |  | Legs | ND | 26.19 | D | ND | ND | 72.75 | 19.87 | ND |
|  |  |  | Tail | 3.9 | 121.9 | 2.28 | ND | ND | 22.07 | 6.01 | ND |
|  |  |  | Brain | 19.63 | 177.3 | 5.97 | ND | ND | 33.01 | D | ND |
|  |  |  | Claw | ND | 31.5 | 2.48 | ND | ND | 25.77 | 5.55 | ND |
|  |  |  | Claw | 4.76 | 39.44 | D | ND | ND | 16.71 | D | ND |
| 2022 | L5 | Female | Eyeballs | 2.05 | 127.7 | D | D | ND | 20.08 | D | ND |
|  |  |  | Legs | D | 122.8 | D | D | ND | 13.52 | D | ND |
|  |  |  | Tail | 7.14 | 603.5 | D | 9.17 | ND | 39.19 | 10.73 | ND |
|  |  |  | Brain | ND | 118.6 | 5.18 | 5.53 | D | 129.3 | 17.11 | ND |
|  |  |  | Claw | 3.67 | 193.7 | D | D | D | 4.83 | D | ND |
|  |  |  | Claw | 2.73 | 195.1 | D | D | D | 15.91 | D | ND |
|  |  |  | Eggs | 20.77 | 557 | D | 16.97 | ND | 13.54 | 15.36 | ND |
| 2022 | L6 | Male | Eyeballs | 2.53 | 88.79 | D | D | ND | 11.45 | D | 6.5 |
|  |  |  | Legs | D | 93.8 | D | D | ND | 15.17 | D | ND |
|  |  |  | Tail | 18.03 | 496.8 | D | 25.45 | ND | 27.8 | D | D |
|  |  |  | Brain | 19.53 | 157.5 | D | D | D | 17.93 | 22.69 | ND |
|  |  |  | Claw | 1.9 | 172.8 | D | D | ND | 16.24 | D | ND |
| 2022 | L7 | Female | Eyeballs | 1.86 | 146 | D | D | D | 27.88 | D | D |
|  |  |  | Legs | D | 150 | D | D | ND | 18.8 | D | ND |
|  |  |  | Tail | 2.79 | 339.4 | D | D | D | 78.19 | D | 8.88 |
|  |  |  | Brain | 15.56 | 228.6 | D | D | D | 12.03 | D | ND |
|  |  |  | Claw | 1.52 | 119.4 | D | D | ND | 15.89 | D | ND |
|  |  |  | Claw | 1.32 | 107.1 | D | D | D | 15.68 | D | D |
|  |  |  | Eggs | 14.01 | 457 | D | 11.96 | ND | 8.75 | D | ND |
| 2022 | L8 | Female | Eyeballs | D | 87.23 | D | ND | ND | 17.92 | D | D |
|  |  |  | Legs | D | 135.9 | D | D | ND | 17.28 | D | ND |
|  |  |  | Tail | 2.23 | 864.6 | D | 16.93 | ND | 18.82 | D | D |
|  |  |  | Brain | 2.76 | 139.6 | D | D | ND | 15.15 | D | ND |
|  |  |  | Claw | D | 209.1 | D | D | D | 13.6 | 11.85 | ND |
|  |  |  | Claw | 1.71 | 178 | D | 4.29 | D | 13.02 | D | ND |
|  |  |  | Eggs | 3.07 | 623.4 | D | 15.24 | ND | 7.21 | 12.02 | D |
